# Video decision support tool promoting values conversations in advanced care planning in cancer: protocol of a randomised controlled trial

**DOI:** 10.1186/s12904-021-00794-3

**Published:** 2021-06-24

**Authors:** Natasha Michael, Clare O’Callaghan, Ekavi Georgousopoulou, Adelaide Melia, Merlina Sulistio, David Kissane

**Affiliations:** 1 Supportive, Psychosocial and Palliative Care Research Department, Cabrini Health, Melbourne, VIC Australia; 2 School of Medicine, Sydney Campus, University of Notre Dame Australia Darlinghurst, Darlinghurst, NSW Australia; 3 Faculty of Medicine, Nursing and Health Sciences, Monash University, Melbourne, VIC Australia; 4 Departments of Psychosocial Cancer Care, St Vincent’s Hospital, Sydney, NSW Australia

**Keywords:** Advance care planning, Cancer, Communication, End-of-life care, Complex intervention, Decision aid

## Abstract

**Background:**

Views on advance care planning (ACP) has shifted from a focus solely on treatment decisions at the end-of-life and medically orientated advanced directives to encouraging conversations on personal values and life goals, patient-caregiver communication and decision making, and family preparation. This study will evaluate the potential utility of a video decision support tool (VDST) that models values-based ACP discussions between cancer patients and their nominated caregivers to enable patients and families to achieve shared-decisions when completing ACP’s.

**Methods:**

This open-label, parallel-arm, phase II randomised control trial will recruit cancer patient-caregiver dyads across a large health network. Previously used written vignettes will be converted to video vignettes using the recommended methodology. Participants will be ≥18 years and be able to complete questionnaires. Dyads will be randomised in a 1:1 ratio to a usual care (UC) or VDST group. The VDST group will watch a video of several patient-caregiver dyads communicating personal values across different cancer trajectory stages and will receive verbal and written ACP information. The UC group will receive verbal and written ACP information. Patient and caregiver data will be collected individually via an anonymous questionnaire developed for the study, pre and post the UC and VDST intervention.

Our primary outcome will be ACP completion rates. Secondarily, we will compare patient-caregiver (i) attitudes towards ACP, (ii) congruence in communication, and (iii) preparation for decision-making.

**Conclusion:**

We need to continue to explore innovative ways to engage cancer patients in ACP. This study will be the first VDST study to attempt to integrate values-based conversations into an ACP intervention. This pilot study’s findings will assist with further refinement of the VDST and planning for a future multisite study.

**Trial registration:**

Australian New Zealand Clinical Trials Registry No: ACTRN12620001035910. Registered 12 October 2020. Retrospectively registered.

**Supplementary Information:**

The online version contains supplementary material available at 10.1186/s12904-021-00794-3.

## Background

All people have the right to make decisions about how they are cared for in the future. However, many lose this ability as they approach the end-of-life (EOL). Advance care planning (ACP) may provide an avenue to ascertain patients’ wishes in circumstances where they are unable to articulate them due to a loss of capacity [1, 2]. A recent multidisciplinary panel of ACP experts defined ACP “as a process that supports adults at any age or stage of health in understanding and sharing their personal values, life goals, and preferences regarding future medical care” [3]. Elucidating ACP enhances desirable EOL outcomes, including patient-caregiver confidence and satisfaction [4, 5], quality of death [2, 6], and family bereavement experiences [6]. ACP requires reflection and communication and is an iterative process that is both individualized and shared [7, 8]. Contemporary views have shifted from ACP focused solely on treatment decisions at the EOL and medically orientated advanced directives. It instead encourages conversations that communicate personal values and life goals to support patient-caregiver communication and decision-making [9], family preparation [10, 11], and the actualization of ACP’s through innovative interventions [12].

Specific ACP challenges are recognized in cancer care, with ACP in cancer limited in completion, scope, timing, and translation into desired care [13]. Patients commonly prefer discussions later in their cancer journey [14], initiated by their treating physicians, and prefer multiple opportunities for conversations [13, 15]. The physician is required to strike a balance between engendering hope and maintaining truthful communication that ideally incorporates prognostic information [16]. Low uptake may be associated with standardized programs failing to capture the complex social and emotional nuances experienced by the cancer patient and their family across ages, genders, cancer types, and trajectories [17].

Cancer is a family experience, and the heightened involvement of family caregivers in cancer ACP studies is welcomed [10, 18]. Fluctuating awareness of treatment goals among cancer patients and their primary caregivers and the lack of concordance in patient-caregiver communication in cancer impacts effective care [19, 20]. However, cancer care provision in the ambulatory setting has augmented the opportunity to align patient-caregiver communication to support patient-caregiver dynamics, coping, adjustment, and psychological well-being [21]. Patients’ and caregivers’ assertive behaviours alongside caregiver presence in cancer consultations can reinforce patients’ participation in care discussions. This then allows for the triadic alignment of goals between the patient, caregiver, and health professionals [22, 23]. Such opportunities may then allow us to explore how individual and shared family values may influence treatment decision-making.

ACP interventions involving cancer patients on the whole, have increased ACP documentation rates from 15 to 30%–40% but failed to achieve EOL care consistent with patients’ preferences [24]. It is increasingly accepted that patient’s values and beliefs are the best predictors of the choices they make relating to end of life goals and treatment decisions [25], leading to ACP research exploring EOL values and the development of values-based ACP documents [26, 27]. Incorporating values directives into ACP removes the emphasis of decisions on specific medical interventions, such as cardiopulmonary resuscitation and intubation. It allows a focus on questions related to personal and family relationships, future concerns related to health, spiritual care, and end of life contingency planning [26]. In maturing the research around EOL values, tools such as decision aids with designs responsive to diverse philosophical perspectives are needed, with the flexibility to change as patients gain experience with their personal illness course.

Video decision aids or Video Decision Support Tools (VDST) incorporating video vignettes in ACP are garnering considerable interest amongst academics, clinicians, and policymakers due to their ability to dynamically depict diminishing health states and the nature of different treatment options in culturally and ethnically congruent manners. A recent systematic review and meta-analysis of 10 randomised controlled trial’s (RCT’s) (2220 patients) examining VDST to assist ACP found that patients who use a VDST were less likely to indicate a preference for cardiopulmonary resuscitation (pooled RR, 0.50; 95% CI 0.27–0.95) and acquired improved ACP knowledge [12]. Only four trials reported data on completion of advance directives, with no studies examining the effect on improved preparation in decision-making or patient-caregiver communication.

This study aims to build on the paucity of research exploring conversations about individual values between cancer patient-caregiver dyads and examining their impact on EOL decision-making and ACP. We hypothesize that patients exposed to a VDST that models values-based ACP discussions between patient-caregiver dyads can be an innovative approach to promote ACP in cancer. This study aims to evaluate the effect of a VDST depicting values communication on rates of completion of ACP, attitudes towards ACP, congruence in communication and preparedness for decision-making. The protocol is outlined according to the SPIRIT (Standard Protocol Items: Recommendations for Interventional Trials) guidelines [28].

## Methods / design

### Study design and setting

This is a prospective, RCT with two parallel groups receiving usual care (UC) or VDST intervention forms part of a research program of ACP in cancer developed in accordance with the Medical Research Council framework for developing complex interventions [29]. In this exploratory study, participants are enrolled in the study as dyads – a patient diagnosed with incurable cancer and a nominated caregiver. In this study, caregivers are defined as a relative, partner, or friend who has a significant relationship with the patient and provides them with social, psychological, and physical assistance [30].

The study will be conducted across three sites in a large not for profit health network Melbourne, Australia. ACP in the hospital is governed by legislation through a jurisdictional Medical Treatment Planning and Decision Act 2016 [31]. The Act establishes a single framework for medical treatment decision-making for people without decision-making capacity that ensures that people receive medical treatment consistent with their preferences and values. Standard forms under the Act allow for the appointment of a Medical Treatment Decision Maker (MTDM) and Support Person and completion of a Values and Instructional Directive (Table 1).

**Table 1:** Components of an advance care plan/directive

**Appointment of Medical Treatment Decision Makers and Support Persons**	
Medical Treatment Decision Maker*	A medical treatment decision maker has the legal authority to make medical treatment decisions on behalf of the patient if they do not have the decision-making capacity to make a decision. It is the first person the patient listed who is reasonably available, and willing and able to make a decision. Only adults can appoint a medical treatment decision maker.
Support Person	A support person can access, or help a patient access health information relevant to their medical treatment. The support person does not have the power to make medical treatment decisions on the patient’s behalf.
Medical Enduring Power of Attorney	A medical enduring power of attorney authorizes another person to make decisions about medical care and treatment on a patient’s behalf if they do not have the decision-making capacity to make a decision. The person making the medical enduring power of attorney is called the appointer, and the person who accepts the appointment is the agent.
**Values and Instructional Directives**	
Values Directive	A medical treatment decision maker is legally required first to consider the patient’s values directive when making decisions about their medical treatment.
Instructional Directive	An instructional directive is legally binding and communicates the patients’ medical treatment decision(s) directly to their health practitioner(s). It is recommended that the patient consult a medical practitioner if they choose to complete an instructional directive. • An instructional directive will only be used if the patient does not have the decision-making capacity to make a medical treatment decision. • The medical treatment decisions in the instructional directive take effect as if the patient has consented to, or refused to, begin or continue medical treatment. • If any of the statements in an Instructional Directive are unclear or uncertain in particular circumstances, it will become a values directive. • In some limited circumstances set out in the Act, a health practitioner may not be required to comply with an instructional directive. • The patient has the option of consenting to or refusing future medical treatment.

*The appointment of a MTDM replaced the appointment of a Medical Enduring Power of Attorney (MEPOA). A MEPOA appointment made before the law changed is recognized under the new Act

### Participants and recruitment

Patients with non-curable cancer will be recruited from the oncology and palliative care across different sites at the health network. Eligible patients will have a diagnosis of incurable cancer and not have completed current ACP documents. Patients who have previously only appointed a MEPOA will be eligible to participate. Both patients and caregivers will have to be over the age of 18, be sufficiently proficient in English, and have a clinically determined prognosis of a minimum of 8 weeks post-randomization. Patients and caregivers will be ineligible for the trial if they are deemed too unwell and are unable to give informed consent due to cognitive or language barriers.

### Development of video vignettes

The video vignettes will be developed from the findings of our preliminary research into ACP in cancer patients [7, 10]. These studies incorporated the use of written vignettes, depicting a cancer patient’s scenarios across four stages of the cancer trajectory. In our preliminary studies, patients were depicted as declining in function and requiring various levels of care [7, 10, 32, 33]. Vignettes were presented to patients and caregivers in interviews and focus groups to elicit views on ACP and subsequently to patient-caregiver dyads as part of a facilitator-guided intervention in a feasibility study [32]. Qualitative secondary analysis on the use of written vignettes revealed that the vignettes provide a platform to promote values-based conversations and may facilitate congruence in communication between the patient and caregiver [33].

In developing the video vignettes, the research team will adhere to suggested guidelines for the preparation of scripted video vignettes which are 1) deciding on the appropriateness of the use of video vignettes, 2) developing a valid script, 3) designing valid manipulations, 4) converting written scripts to video, 5) administering the videos [34].

The written script will be developed by senior clinicians, drawing from cases used in previous studies [7, 10]. Manipulations will include representation of patient-caregiver dyads from differing ages, gender, stages of illness and relationships e.g. older patient and spouse/partner; middle-aged patient and sibling; younger patient and friends). This, as well as settings and furnishing, will be selected to enhance the participant’s perception of reality [34]. The video vignettes will depict dyads scenarios communicating across three stages of a cancer trajectory, with each stage introducing considerations for completion of different sections of an ACP (Table 2). Professional actors will be sourced from known professional college and university sources, and a pilot video will be created before the filming of the final videos. The video vignettes will be circulated to a multidisciplinary team for validation and approval and further editing before the final video intervention is created.

**Table 2:** Predicted scenarios depicted in the video decision support tool

	Cancer stage	Values conversation depicted	ACP outcomes encouraged
**Vignette 1** **Early cancer diagnosis**	Early diagnosis, good performance status	Active treatment to preserve life at any cost	Appointment of a Medical Treatment Decision Maker and Support Person
**Vignette 2** **Living with serious illness**	Progressive metastatic disease, deteriorating performance status	Sustaining a reasonable quality of life through illness. Reflection of values and reprioritization of life choices	Completion of Values Directive. Consider when to discontinue cancer treatments / accept or refuse treatments based on acceptable quality of life
**Vignette 3** **Approaching the end of life**	Advanced metastatic disease, increased dependence	Effects of progressive frailty on quality of life despite active treatment. Reflection of meaningful relationships and EOL values	Completion of Instructional Directive. Discuss cardiopulmonary resuscitation, invasive/life-prolonging interventions, preferred place of death, contingencies e.g. funeral plans, spiritual needs.

### Study procedures

**Fig. 1:**
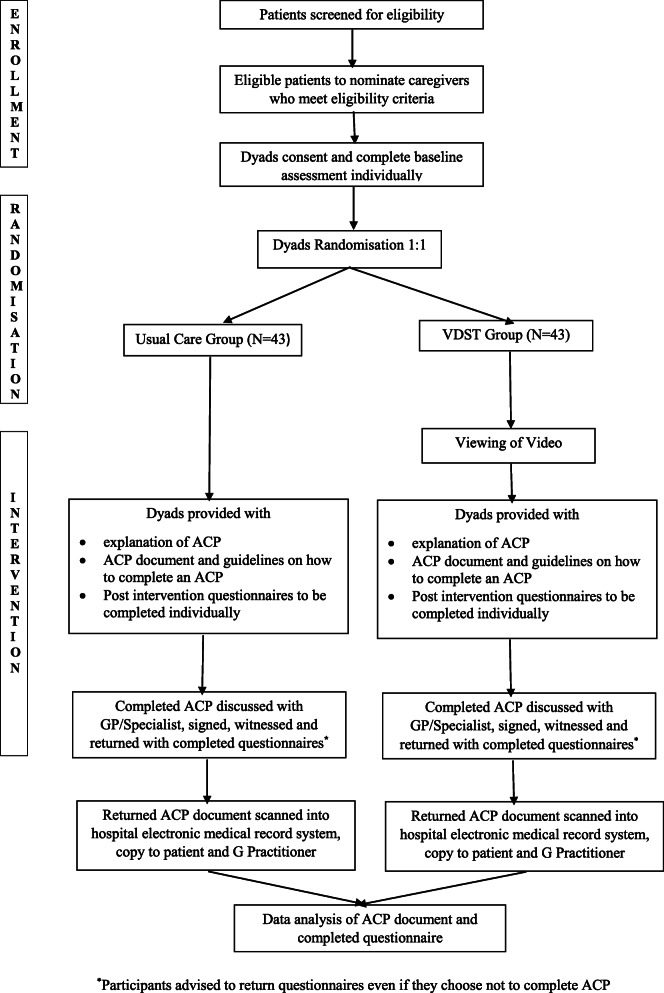
Study procedure

The study procedure is shown in Fig. 1. Participating clinicians will screen patients for eligibility, and interested eligible patients will be asked to nominate a participating caregiver. Eligible dyads will be informed verbally and in writing about the study and will be invited to meet with a research facilitator in their own homes or an allocated room in the hospital at an assigned date and time.

Patients and caregivers will be consented individually by the research facilitator and will complete individual baseline pre-questionnaires (Supplementary file). Participants will then be randomised as dyads to either the UC or VDST arm. All participants will be advised that participation is voluntary, they may withdraw at any time, and that all information gathered will be treated in the strictest confidence. Patients and caregivers will also be advised that non-participation will not affect their care or their choice to complete an ACP.

Patients from both groups will be advised to discuss their completed ACP with their general practitioner or treating specialist to clarify any questions. The completed ACP will have to be signed, witnessed and returned with the post-questionnaire in a stamped return envelope. Participants will be advised to return the questionnaires even if they choose not to complete the ACP. Participants will receive two telephone call prompts at two-week intervals on completion of the intervention as a reminder to return the completed documents. Returned ACP’s will be scanned into the hospital’s electronic record system, and a copy returned to the patient.

### Sample size and power

Based on the primary outcome of the completion of ACP documentation and assuming a completion rate of around 37% for the control group, with a total of 86 participants/dyads (43 per group) we will be able to show a significant improvement to 67% completion for the VDST group (power of 80%, two sided 5% significance level). Assuming an estimated dropout rate of 30%, recruitment of 112 dyads will be required. Based on previous published studies, we felt that a sample of this size would give us sufficient methodological experience to conduct a subsequent fully powered study [12].

### Quality standards

#### Randomization and blinding

Randomization occurs after completion of baseline assessments. An independent randomization administrator will complete randomization at the individual level using a computer-generated random number sequence in blocks of 6 in 1:1 ratio and with concealed allocation using numbered envelopes. The nature of the intervention makes it impossible to blind study participants. Treatment group assignments will therefore be non-blinded, but outcome assessors will be blinded to allocation.

#### Facilitator training and distress management

Research facilitators will be trained in ACP principles and familiarised with recent changes to legislation with the introduction of the Medical Treatment Planning and Decision Act 2016 [31]. They will additionally be familiarised with ACP and the specificities of the appointment of an MTDM and completion of Instructional and Values Directives. Given that the video vignettes may potentially elicit distress in participants, research facilitators will also be trained on how to respond to distress and procedures around seeking additional support for participants. Researchers will be advised to offer to discontinue the video if deemed appropriate.

#### Data monitoring and confidentiality

Research facilitators will be asked to complete field notes as part of the study monitoring plan. The project team will meet monthly to review the progress of the study. Adherence to the research protocol will be monitored throughout the study. Protocol violations or operational issues will be discussed and resolved at project team meetings. The study steering committee will monitor the study and provide ongoing oversight into early results. If necessary, modifications to the study will be made. To ensure confidentiality, data will be stored in a secure database. Information and measurements will be stored independently from identifiable personal information.

### Study arms

#### Usual care

Patient-caregiver dyads will be provided with a verbal explanation of ACP, an ACP document, and verbal and written guidelines on how to complete the document. Questions will be fielded, and finally, participants will be provided with the patient and caregiver post-questionnaires to be completed individually and returned with the completed ACP in a stamped return envelope.

#### VDST group

Participants randomised to the VDST group will be shown the video on a mobile computer. The video will be viewed by patient-caregiver dyads simultaneously. Research facilitators will offer to discontinue the video at any point if it elicits distress. Following this, the participants will be provided with a verbal explanation of ACP and be provided with the hospital’s ACP document, and verbal and written guidelines on how to complete the document. Questions will be fielded, and finally, participants will be provided with patient and caregiver post-questionnaires to be completed individually and returned with the completed ACP in a stamped return envelope.

### Outcome assessment

The primary outcome measure will be the completion of ACP documentation. We will specifically examine the completion of various sections of the ACP (Appointment of MTDM and Support Person, Values and Instructional Directives). The following secondary outcomes that will be assessed will be attitudes towards ACP, congruence in communication and preparedness for decision-making*.*

#### Data collected and measures used

Table 3 outlines the measures used at baseline and post-intervention. The baseline questionnaire will include the patient’s and caregiver’s demographic information, including age, sex, marital status, place of birth, the relationship between patient and caregiver, primary cancer diagnosis, and length of time living with the diagnosis. Patients will be asked if they had previously discussed prognosis with their doctor.

**Table 3:** Measures used pre and post UC and VDST

Measures used	Patient		Caregiver	
	Baseline: Pre UC/VDST	Post UC/VDST	Baseline: Pre UC/VDST	Post-UC/VDST
Demographics	**×**		**×**	
DASS-21	**×**			
ACP Attitudes	**×**	**×**	**×**	**×**
CCAT	**×**	**×**	**×**	**×**
PDMS	**×**	**×**	**×**	**×**

*DASS-21* Depression, Anxiety and Stress Scale, *CCAT* Cancer Communication and Assessment Tool, *PDMS* Preparation for Decision Making Scale

The following variables and outcomes will be assessed:

1. *Baseline Depression, Anxiety and Stress scores*. The DASS 21 scale is a validated 21 item self-reported questionnaire designed to measure the negative emotional states of depression, anxiety, and stress [35]. Evaluation of the DASS 21 in cancer has shown acceptable internal consistency reliability for the Depression subscale (α = .90) and Anxiety subscale (α = .70) with construct validity to measures of suicidal ideation, quality of life, self-rated health, and depressed mood [36].
2. *Attitudes towards ACP*. Understanding of, opportunities, distress, and confidence related to ACP will be assessed using a previously designed and tested questionnaire comprising nine patient and eight caregiver items, measured on a 10 point Likert scale [32].
3. *Congruence in decision-making* will be measured using the Cancer Communication Assessment Tool for Patients and Families (CCAT-PF). CCAT-PF consists of 18 items and measures congruence in patient-caregiver communication with the analogous patient (CCAT-P) and family (CCAT-F) instruments, exploring preferences, values, and experiences in treatment decision-making. The CCAT-PF demonstrated internal reliability coefficients for the CCAT-P (α = .52), CCAT-F (α = .50), and CCAT-PF (α = .60). Higher CCAT-PF scores are significantly correlated with greater patient depression and perceived family conflict, lower patient-caregiver assessment and well-being, and less expressiveness and family cohesion [37].
4. *Preparation for decision-making* will be measured using the Preparation for decision-making scale, a validated scale assessing patient and caregiver perception of an intervention’s usefulness. Psychometric analysis has shown Alpha coefficients for internal consistency ranging from 0.92 to 0.96 and that the scale discriminated significantly between patients who did and did not find a decision aid helpful (*p* < 0.0001) [38].

## Statistical analysis

Descriptive statistics will be presented as frequencies and relative frequencies for categorical variables, mean and standard deviation for normally distributed continuous variables, or median and interquartile range for continuous variables that are not normally distributed. We will examine secondary outcomes between the two groups comparing pre and pre and post-test intervention changes with Chi-square or Fisher’s exact test for categorical variables and two-sample *t*-tests or Mann-Whitney test for continuous variables.

Additionally, multiple linear regression will be performed, addressing the effect of socio-demographic and clinical variables to explore factors associated with the rates of completion of ACP All results obtained will be presented at a confidence interval of 95%. Thus *p* < 0.05 is assumed to be statistically significant. The statistical software SPSS 23 will be used in this analysis.

## Discussion

We present the protocol for a pilot randomised control trial developed as part of a body of work exploring ACP in cancer in the Australian setting. This study follows from previous studies conducted by members of the research team, demonstrating the feasibility and acceptability of the written vignette technique, recruitment of patient-caregiver dyads, and completion of ACP’s. The use of video vignettes as a decision aid is supported by a Cochrane review of 115 RCTs involving 34,444 participants showing that compared to UC, decision aids improve knowledge, accurate risk perception, likelihood of selecting options congruent with personal values, and reduce decisional conflict [39]. The video vignette technique has been proven to be acceptable and effective in several North American settings and, it is possible that this equally effective in the Australian setting [12].

A novel aspect of this study is the use of video vignettes to promote values discussion. The use of video to explore a values-based ACP paradigm has been shown to be successful in a single study [40]. This approach’s potential benefit in increasing the uptake of ACP in cancer care may allow for more meaningful EOL care planning between patients and their families. Our use of patient-caregiver dyads is relatively unique to ACP video intervention studies. This is despite evidence that suggests the benefit of congruence in communication between patient and caregiver, particularly in the cancer setting [22].

### Limitations

There remains a paucity of literature on the methodological challenges that may arise with video vignettes’ development. Even the most realistic scripted video vignettes may differ from communication as it naturally unfolds. It is suggested that the manipulation of less defined concepts such as ‘the communication of values’ in our study through video vignettes poses specific challenges due to challenges in operationalizing such concepts.

Intervention studies in cancer cohorts typically demonstrate significant attrition rates due to progressive illness and high mortality [41]. Cancer patients also not uncommonly remain ambivalent or choose to relinquish or reject ACP as they potentially elicit death anxiety or other existential distress forms. Finally, this study provides an intervention at a single time point, is restricted to a single site and will not measure subsequent congruence between documented ACP decisions and EOL outcomes. It also limits recruitment to English-speaking participants with no provision made for those with limited health literacy and cannot participate.

## Conclusion

We need to continue to explore innovative ways to engage cancer patients in ACP. This pilot study’s findings will assist with further refinement of the VDST and planning for a future multisite study.

## Supplementary Information

**Additional file 1.**

## Data Availability

Not applicable. Data sharing is not applicable as this article has no datasets that have been generated or analysed yet. The datasets generated during the study will be available from the corresponding author on reasonable request.

## Abbreviations

ACP: Advance care planning;
CCAT: Cancer Communication Assessment Tool
CCAT-P: Cancer Communication Assessment Tool for Patient
CCAT-F: Cancer Communication Assessment Tool for Families
CCAT-PF: Cancer Communication Assessment Tool for Patient and Families
EOL: End-of-life
MEPOA: Medical Enduring Power of Attorney
MTDM: Medical Treatment Decision Maker
UC: Usual care
VDST: Video decision support tool
