# Supplementary material for: Video decision support tool promoting values conversations in advanced care planning in cancer: protocol of a randomised controlled trial
Source: BMC Palliat Care. 2021 Jun 24;20:95. doi: 10.1186/s12904-021-00794-3 (PMC8229383; doi:10.1186/s12904-021-00794-3)
Supplement: Supplementary file 1 — Additional file 1. [file 12904_2021_794_MOESM1_ESM.pdf]

UR No.:

Surname:

Given Name:

D.O.B.:

Please fill in if no patient label available

MY ADVANCE CARE DIRECTIVE

This Advance Care Directive replaces any previous directives that I may have made. I understand that my values and beliefs may be different from those of the people I choose to make decisions for me and I ask them to make the decisions which I would make. I also understand that I can change my Advance Care Directive at any time.

This Advance Care Directive is in effect only when I do not have capacity to make my own decisions.

I have made this Advance Care Directive after discussion with the following health care providers (e.g. doctors, nurses, social workers, etc):

Name: *Role:*

Name: *Role:*

Name: *Role:*

Why?

Have your voice heard

It is good to think about your future health care needs and to discuss these with others.

If a time comes when you are unable to make your own decisions, someone else will be asked to make decisions on your behalf. You can choose who that person is and help that person to represent you by telling them what would be important to you at this stage in your life. This document suggests some of the issues you might like to discuss with both your doctor and the person you have chosen (or who will be chosen) to represent you.

The document is in four parts:

Part 1: choose who you trust to speak on your behalf and who you want to have access to your medical information

Part 2: express the values that are important to you to help your decision-maker

Part 3: give binding instructions about certain medical situations

Part 4: obtain signatures of witnesses to the creation of this document

You may complete all or part of Part 1, 2 and 3.

Part 4 must be completed for this Directive to be valid.

Me

About me

Name:

Date of birth: / /

Address:

MY ADVANCE CARE DIRECTIVE

UR No.:

Surname:

Given Name:

D.O.B.:

Please fill in if no patient label available

Part 1 – Appointment of Medical Treatment Decision Makers and Support Person

**Who?** **Who will speak for me?**	This section allows you to document one or two people who you know well and trust to make health decisions in the event that you are unable to do so for yourself. The second person you list will only be asked if the first person is unavailable.<br>You can also appoint a Support Person who will help you make decisions while you still have decision-making capacity – they will have access to your medical information. Your Support Person can also be your Medical Treatment Decision Maker.<br>The people you select to be your Medical Treatment Decision Makers and Support Person must sign this page to accept the role.

Medical Treatment Decision Maker 1

Name:	
Address:	
DOB:	

Acceptance of appointment: I understand the obligations of an appointed Medical Treatment Decision Maker and undertake to act in accordance with any known preferences and values of the person making the appointment. I undertake to promote the personal and social wellbeing of the person making the appointment and have read and understand any advance care directive that the person has given.

Name: Signature: Date:

Medical Treatment Decision Maker 2

Name:	
Address:	
DOB:	

Acceptance of appointment: I understand the obligations of an appointed Medical Treatment Decision Maker and undertake to act in accordance with any known preferences and values of the person making the appointment. I undertake to promote the personal and social wellbeing of the person making the appointment and have read and understand any advance care directive that the person has given.

Name: Signature: Date:

Support Person

Name:	
Address:	
DOB:	

Acceptance of appointment: : I understand the obligations of an appointed Support Person and undertake to act in accordance with any known preferences and values of the person making the appointment. I undertake to promote the personal and social wellbeing of the person making the appointment and have read and understand any advance care directive that the person has given

Name: Signature: Date:

Witnesses to appointment of Medical Treatment Decision Makers and/or Support Person: I certify that the person making the above appointments and the person/people accepting those appointments appear to have decision-making capacity and have freely and voluntarily signed the document

Witness One: Authorised witness

Name:

Signature: Date: / /

Qualification:

Witness Two:

Name:

Signature: Date: / /

MY ADVANCE CARE DIRECTIVE

UR No.:

Surname:

Given Name:

D.O.B.:

Please fill in if no patient label available

Part 2 – Values Directives

**What?** **What is important to me?**	We are all unique and have different beliefs, values and goals. Here you can say what is important to you. What does it mean to you to 'live well'? This information will be used by people making decisions for you to help them make the decisions that you would have made yourself. These statements are a guide to treatment decision making only. If you wish to legally refuse treatment, see Part 3 of this form.

1. I am currently receiving care and treatment for the following health conditions:

2. What is difficult for me to do now because of my health conditions?

3. What worries me about what will happen to my health in the future?

4. The following things worry or concern me unrelated to my health. For example: family concerns, hopes and fears, emotional issues, accommodation, people I do not want involved.

5. These are the things in life that have a lot of meaning for me. (For example: enjoying activities, spiritual or religious beliefs, family or friends, pets, reading books, independence.)

UR No.:

Surname:

Given Name:

D.O.B.:

Please fill in if no patient label available

Part 2 – Values Directives continued

6. The following things are important to me and they may help my Medical Treatment Decision Maker to make medical decisions in the future for me (for example: special religious or cultural needs):

7. If I have an illness or injury and can no longer make treatment decisions for myself, I would most likely agree to *(Please tick one box only)*:

- ☐ Any treatments that will make me live longer and are suitable for my medical condition (even if they could cause me distress)

Or

- ☐ To be kept comfortable and be provided with treatments that are not distressing and mainly treatments aimed at relief of pain and other symptoms

Or

- ☐ For my Medical Treatment Decision Maker to decide what is best for me, based on what the doctors tell them about me at that time.

8. Other things that are important to me are:

9. If I am nearing death, the following things are important to me (e.g. where I would prefer to die, spiritual/faith rituals or requests, who I would like with me, funeral preferences)

Cabrini

MY ADVANCE CARE DIRECTIVE

UR No.: __________

Surname: __________

Given Name: __________

D.O.B.: __________

Please fill in if no patient label available

Part 3 – Instructional Directives

What?

What treatment decisions have you already made?

If you wish your Medical Treatment Decision Maker and healthcare providers to be able to take into account your circumstances when you are not able to make decisions, you should use **Part 1** and **Part 2** of this form only. **Part 4** must also be completed

In **Part 3** you can make binding statements called 'Instructional Directives' that will communicate your medical treatment decision directly to your healthcare providers.

It means that no-one else will be asked to make decisions about those treatments for you. It is very important that you consult your health practitioner if you choose to

complete an Instructional Directive, in order to ensure that it is written in a way that can be safely interpreted. If your Instructional Directives are unclear, they will still be considered as descriptions of your values.

To give more general guidance to your Medical Treatment Decision Maker see Part 2 of this form. **Cross out this page if you do not wish to consent to or refuse any specific medical treatments.**

10. **Cardiopulmonary Resuscitation (CPR) involves chest compressions and artificial ventilation to manually save brain function, blood circulation and breathing for someone in cardiac arrest. These interventions are used when a person's heart stops beating, and they may or may not restore life.**

If my heart stops beating:

☐ Attempt resuscitation if clinically indicated

Or

☐ Do NOT attempt resuscitation

Comment: __________

____

11. **Organ donation:** Very few people die in a way that allows them to be considered for organ donation. One organ donor can save or improve the lives of many others. Your family will be asked to confirm your consent for donation.
In the event that I am able to be considered for organ, eye and/or tissue donation when I die, I wish to be a donor:

☐ Yes ☐ No

12. Here you can write other **Instructional Directives**. Keep in mind that these should include details about the circumstances in which the directive will apply, as well as specifics of the treatments to which you either give or refuse consent e.g. "If I am ever suffering from (*insert details of condition*), I give my consent to/refuse (*insert details of treatment*).

____

____

____

____

____

Cabrini

MY ADVANCE CARE DIRECTIVE

UR No.: ________________

Surname: ________________

Given Name: ________________

D.O.B.: ________________

Please fill in if no patient label available

Part 4 – Witnessing and signatures

Your Declaration:

I make this Advance Care Directive and any appointments within it freely and voluntarily, and I understand the nature and effect of each statement within the Directive.

Name: ________________

Signature: ________________ Date: / /

Witnesses to the signing of this Advance Care Directive: I certify that the person giving this Advance Care Directive appears to have decision-making capacity and has freely and voluntarily signed the document in the presence of two witnesses, neither of whom has been appointed as a Medical Treatment Decision Maker. The person appears to understand the nature and effect of all statements made within this document.

**Witness One: Registered Medical Provider**	**Witness Two:**
Name:	Name:
Signature: Date: / /	Signature: Date: / /
Qualifications:	

Sharing your Advance Care Directive

I understand that it is important to discuss these healthcare preferences with my GP, Medical Specialists, local hospital, my family / friends and particularly my Medical Treatment Decision Makers. I have discussed and provided a copy of My Advance Care Directive to the following people:

Name	Contact Phone Number

Instructions for completing your **Cabrini Advance Care Directive**

Cabrini

This document provides information to assist you in completing your Cabrini Advance Care Directive (ACD). You can download and print the Advance Care Directive form from the Cabrini website: www.cabrini.com.au/advancecareplanning

If you need assistance completing the form, you can contact

- your General Practitioner,
- your Medical Specialist, or
- Cabrini Social Work department Ph: 9508 1222 (switchboard) and ask for Social Work.

Who can complete the form?

Advance Care Directives are often undertaken when someone has a serious illness or is of advancing age. However, they can be made at any time. Younger, healthy people will benefit from completing an Advance Care Directive. Advance Care Directives are strongly recommended for people who meet the following criteria:

- Have severe illnesses that might in time lead to death, such as advanced kidney, lung or heart failure, cancer, progressive neurological disease or early dementia
- Residents being admitted to residential aged care
- Aged over 75 years
- Have multiple medical problems causing increasing frailty or reduced ability to perform usual activities
- Have clear wishes about what sort of medical treatment they would want if they were ever unable to make their own decisions.

In order to complete a valid Advance Care Directive, two witnesses, one of whom is a doctor, must sign to confirm that you have the capacity to understand all of the statements within the document. Neither witness can be someone you have appointed as your Medical Treatment Decision Maker.

A person under 18 years of age is able to make an Advance Care Directive and appoint a Support Person if they have the decision-making capacity to do so. However, only people over 18 years of age are able to appoint a Medical Treatment Decision Maker.

What is in the form?

There are 4 parts to the Advance Care Directive form:

1) Appointment of Medical Treatment Decision Maker and Support Person
2) Values Directives
3) Instructional Directives
4) Witnessing and signatures

You can complete all or part of Parts 1-3. Part 4 must be completed for Parts 2 & 3 to be valid.

You should draw a line through any sections of the Advance Care Directive form that you choose not to fill out.

This Advance Care Directive will automatically replace any earlier directives you have made, and the appointment of a Medical Treatment Decision Maker will replace any previously appointed Medical Enduring Powers of Attorney. It is recommended that you cancel previous directives or appointments by striking a line through each page and writing "cancelled" or "replaced" on all copies of the earlier forms.

If you have them available, you can use hospital patient identification stickers in the top right hand corner of each page of the document – you can ask Cabrini staff for the stickers. Otherwise you should write your name and date of birth on each page of this document.

Completing the form

The page numbers below refer to the page in the Advanced Care Directive (ACD) form.

PAGE ONE (of the ACD form)

Me: Make sure you fill in your own details at the bottom of page one.

PAGE TWO (of the ACD form)

Who: Appointment of Medical Treatment Decision Maker(s) and Support Person

Medical Treatment Decision Maker

Your Medical Treatment Decision Maker is the person who will be asked to make medical decisions for you if you are ever unable to. The first person on the hierarchy below who is available and willing to act in the role, and who has a close and continuing relationship with you, will become your Medical Treatment Decision Maker. If you appoint someone, they will be at the top of the hierarchy. It is worthwhile appointing your Medical Treatment Decision Maker, even if you are appointing someone on the hierarchy; this can make it easier for your family and friends to be clear about who you want to make decisions for you and can avoid confusion and uncertainty for them.

Medical Treatment Decision Maker Hierarchy:

1) A person appointed by you
2) A guardian appointed by VCAT
3) Your spouse or domestic partner
4) Your primary carer (not including paid carers or service providers)
5) Your oldest adult child
6) Your oldest parent
7) Your oldest adult sibling
8) The Public Advocate

If none of your appointed Medical Treatment Decision Makers or relatives are available, the Public Advocate will be asked to make medical decisions for you.

If you appoint a Medical Treatment Decision Maker using this form, any previous Medical Enduring Powers of Attorney that you have appointed will be replaced by the person/people you appoint with this form. If you have previously appointed a Medical Enduring Power of Attorney, it is the same as appointing a Medical Treatment Decision Maker with this form.

Only adults are able to appoint a Medical Treatment Decision Maker.

Using this form, you can appoint up to two Medical Treatment Decision Makers – the person listed second will only be approached if your first choice is unavailable or unwilling to act as your Medical Treatment Decision Maker.

The name, address and date of birth for your chosen Medical Treatment Decision Maker(s) must be completed.

The people you appoint as Medical Treatment Decision Maker must sign the form to say that they are willing to accept the appointment.

Support Person

Your Support Person's role is to help you make decisions and also to help your Medical Treatment Decision Maker to make decisions on your behalf if you are ever unable to do so. Your Support Person will not be asked to make decisions on your behalf. By appointing someone as your Support Person, you are granting them access to your medical information in order to assist decision making. Your Support Person may be the same person as your Medical Treatment Decision Maker, or a different person.

Witnesses (to appointment of Medical Treatment Decision Maker(s) and/or Support Person)

When you and your appointed Medical Treatment Decision Maker(s) and/or Support Person sign this form, you must do so in the presence of two witnesses who also sign the bottom of this page. It is the role of the witnesses to certify that you have completed this form willingly and without coercion, and that you have the capacity to make the decisions that you and your appointed representatives have made to enter into this agreement.

One of the witnesses must be an **Authorised Witness**; that is someone who is registered in specific occupations, including the following: dentist, legal practitioner, medical practitioner, nurse, optometrist, pharmacist, physiotherapist, psychologist, veterinarian, some post office agents, bank officers and clerks of court. Neither witness can be someone you have appointed as your Medical Treatment Decision Maker.

If you appoint a Medical Treatment Decision Maker and/or Support Person, it is recommended that you discuss your Advance Care Directive, and your personal wishes, with that person so that they might understand your thoughts if they are ever needed to act on your behalf. You can also discuss your Advance Care Directive with other family members or friends, and also with your health care providers.

PAGES THREE AND FOUR (of the ACD form)

What: Values Directives

In this section, you can write down information about what is important to you. This information is used by your Medical Treatment Decision Maker to help make decisions if you are ever unable to do so. These statements are not binding; they do not enforce decisions on your Medical Treatment Decision Maker; they simply guide your Medical Treatment Decision Maker to be able to imagine what decisions you would have made for yourself.

- What is important to you?
- What does it mean to live well?
- What things concern you?
- For example, do you want to live for as long as possible? Or are there things that you wouldn't want to live without? If remaining independent is important to you, try to be specific about what it means to be independent, as people have different understandings of what this means. For some people, living independently means being in their own home, while others may be more concerned with being able to take care of their personal grooming, being able to maintain connections with family and friends, or being able to make decisions for themselves
- Do you have specific religious or spiritual beliefs that might affect the type of medical treatment you want?
- Are there other people you would like involved in making decisions about your care?
- Are there things that you would want known if you are nearing death? These may include who you would like with you, where you would most like to be if possible (home, hospital, hospice), music you would like playing, or other spiritual or cultural customs

Questions 1-9 will guide you in what you might like to write in this section.

We encourage you to write Values Directives, as these are very useful in guiding your Medical Treatment Decision Maker and your health care professional when making decisions for you. Recording your wishes and values is important even if you don't appoint a Medical Treatment Decision Maker, as someone will still be asked to consider these statements when making decisions for you; this includes the Public Advocate who will have access to your Advance Care Directive if they are making decisions on your behalf.

PAGE FIVE (of the ACD form)

What: Instructional Directives

In this section, you can make binding statements that will communicate your medical treatment decisions directly to your health care providers. If you make Instructional Directives, it means that you have already made these decisions and your Medical Treatment Decision Maker will not be asked to make these decisions on your behalf. You can use this section to give permission to health care providers to provide certain treatments for you if they are indicated, and you can also use this section to refuse treatments that you do not want.

Questions 10 and 11 relate to two specific examples of Instructional Directives which are commonly considered:

- the use of CPR to attempt to re-start breathing and circulation if they stop, and
- the opportunity to consider being an organ and/or tissue donor after you die.

If you do not wish to answer these questions, you can draw a line through them.

If you do wish to be considered for organ donation, be aware that there are certain medical interventions at the end of your life that are required for donation to be successful. It is recommended that you also register your wishes about organ and tissue donation on the Australian Organ Donor Register. More information about organ and tissue donation can be found on the Donatelife.gov.au website.

Question 12 allows you to add your own Instructional Directives.

It is very important that Instructional Directives are well written. It is strongly recommended they are discussed with your doctor before finalising this form, otherwise it is possible that your directives will be difficult to follow. For example, if you have written "under no circumstances do I wish to be kept alive by a machine that breathes for me"; this means that this treatment will not be provided to you even if it was only required for a short period of time, such as while you have an operation. Your doctor will help you to clarify exactly what it is that you want, and how best to write it to ensure that it is clear.

Each Instructional Directive requires two parts: it is necessary to state the situation in which the directive should apply, and the treatment decision that you have made. It can also be helpful to include the reasons for your statement.

PAGE FIVE (of the ACD form) *continued...*

This will look like:

If I ever have __ *(illness or injury)*__, I do / do not want ___ *(treatment)*____, Because__________________ .

e.g. If I ever suffer a severe injury to my brain and it is thought that I would not be likely to recover from this injury enough to live independently, I do not want to be connected to any machines that will artificially prolong my life. This is because living independently is very important to me.

e.g. If it is required to save my life, I give consent to transfusion of blood and blood products. Although my family are Jehovah's Witnesses, I do not share their religious belief.

If you write an Instructional Directive and it is not clear whether it relates to the condition for which medical treatment decisions need to be made, these statements will be considered Values Directives, and your Medical Treatment Decision Maker will be asked to consider these statements and then to make a decision on your behalf.

Make sure to only write binding instructions in the "Instructional Directives" section of this form, otherwise they will be considered Values Directives only.

Note that if you choose to make an Instructional Directive to refuse CPR in the event that your heart stops beating, you may not receive CPR in an unforeseen emergency (such as drowning or electric shock).

Keep in mind that your health care providers will only provide treatment that is medically appropriate, even if you have consented to it in your Advance Care Directive.

Where an Advance Care Directive contains a statement that would require an unlawful act to be performed or that would require a health practitioner to breach a code of conduct or professional standards, including the Catholic Code of Ethical Standards, these statements are void and have no effect but the remainder of the ACD remains valid.

PAGE SIX (of the ACD form)

Witnessing and signatures - to Advance Care Directive

This page must be completed for your Advance Care Directive to be valid. By signing this page of the form, you are confirming that you have completed the document freely and voluntarily, that is to say that no one has pressured you into writing anything in the directive.

You are also declaring that you understand the implications of all of the statements you have made in the directive. It is highly recommended that you discuss all of the statements within the document with your doctor to ensure it is well written and that you have considered all relevant information.

Your Advance Care Directive must then be signed by two witnesses, one of whom is a doctor. It is recommended that you do this with a doctor you know well; either your GP or your medical specialist. The second witness to the document does not need to be a doctor, but cannot be your Medical Treatment Decision Maker.

In the case of a child making an Advance Care Directive, one of the witnesses must be either a doctor or a psychologist who has completed prescribed training to witness the declaration of an Advance Care Directive of a minor.

The last section of Page 6 allows you to record the people who hold a copy of your Advance Care Directive. You can add to this section every time you distribute your directive to a new person. It is recommended that you keep the original document where you keep other important documents such as wills or passports.

If you are receiving treatment at Cabrini Hospital, a copy should be made and kept in your Cabrini medical record. You can ask for this to happen whenever you are admitted to the hospital, present to the Emergency Department, or visit for outpatient services. It is highly recommended that you give copies of the Advance Care Directive to at least the following people:

- Your Medical Treatment Decision Maker(s) and/or Support Person
- Your General Practitioner
- Your Medical Specialist(s)
- Your local hospital
